# Medaka fish exhibits longevity gender gap, a natural drop in estrogen and telomere shortening during aging: a unique model for studying sex-dependent longevity

**DOI:** 10.1186/1742-9994-10-78

**Published:** 2013-12-23

**Authors:** Singaram Gopalakrishnan, Napo KM Cheung, Bill WP Yip, Doris WT Au

**Affiliations:** 1 State Key Laboratory in Marine Pollution, Department of Biology and Chemistry, City University of Hong Kong, 83 Tat Chee Avenue, Kowloon, Hong Kong SAR

**Keywords:** Lifespan, Aging, Telomerase and telomere, Estrogen profile, Sex difference and medaka *O. latipes*

## Abstract

**Introduction:**

Females having a longer telomere and lifespan than males have been documented in many animals. Such linkage however has never been reported in fish. Progressive shortening of telomere length is an important aging mechanism. Mounting *in vitro* evidence has shown that telomere shortening beyond a critical length triggered replicative senescence or cell death. Estrogen has been postulated as a key factor contributing to maintenance of telomere and sex-dependent longevity in animals. This postulation remains unproven due to the lack of a suitable animal system for testing. Here, we introduce a teleost model, the Japanese medaka *Oryzias latipes,* which shows promise for research into the molecular mechanism(s) controlling sex difference in aging.

**Results:**

Using the medaka, we demonstrate for the first time in teleost that (i) sex differences (female > male) in telomere length and longevity also exist in fish, and (ii) a natural, ‘menopause’-like decline of plasma estrogen was evident in females during aging. Estrogen levels significantly correlated with telomerase activity as well as telomere length in female organs (not in males), suggesting estrogen could modulate telomere length via telomerase activation in a sex -specific manner. A hypothetical *in vivo* ‘critical’ terminal restriction fragment (TRF, representing telomere) length of approximately 4 kb was deduced in medaka liver for prediction of organismal mortality, which is highly comparable with that for human cells. An age conversion model was also established to enable age translation between medaka (in months) and human (in years). These novel tools are useful for future research on comparative biology of aging using medaka.

**Conclusion:**

The striking similarity in estrogen profile between aging female *O. latipes* and women enables studying the influence of “postmenopausal” decline of estrogen on telomere and longevity without the need of invasive ovariectomy. Medaka fish is advantageous for studying the direct effect of increased estrogen on telomere length and longevity without the breast cancer complications reported in rodents. The findings strongly support the notion that *O. latipes* is a unique non-mammalian model for validation of estrogenic influence on telomere and longevity in vertebrates. This laboratory model fish is of potential significance for deciphering the ostensibly conserved mechanism(s) of sex-associated longevity in vertebrates.

## Introduction

Longevity gender gap (LGG) is the longevity difference between the two sexes. Many animals exhibit a longer lifespan in females than males (Table 1). Several theories have been proposed to explain the possible cause(s) of LGG (females > males) [1]. Among them, sex differences in telomere length (longer in females) and estrogen (higher in female) have been given the most attention, particularly in mammalian studies [2-5]. Telomeres are DNA capping structures that protect chromosome ends from recombination and fusion, maintaining genomic stability [6]. Telomeres shorten with age due to inefficiency in DNA replication (a.k.a. end-replication problem) [7]. Shortened telomere length (TL) below threshold level induces cellular senescence or cell death [8]. Progressive erosion of telomere length is an important aging process, which is well recognized by extensive *in vitro* and *in vivo* studies on mammals [4,9]. The enzyme telomerase promotes telomeric repair and reduces telomere erosion by adding conserved repeats of TTAGGG to chromosomal ends [10]. *In vitro* mammalian studies demonstrated that estrogen can stimulate telomerase activity via estrogen-receptor-mediated transcription and post-translational activation of TERT (telomerase reverse transcriptase; the catalytic unit of telomerase) [11,12]. Estrogen is synthesized in all vertebrates and some invertebrates [13,14], therefore estrogen-mediated telomerase activation and telomere maintenance are likely conserved in animals.

**Table 1:** The existence of longevity gender gap (females living longer than males) in different animal taxa

	**Taxa**		**References**
*Vertebrates*	Mammals	Human	[2,3,15-17]
		Chimpanzees	[18,19]
		Gorillas, Orangutans, gibbons, spider monkeys and sifakas	[18,20]
		Rat	[15-17,21]
		Soay sheep, pilot whales and killer whales	[22,23]
	Reptiles	Lizard	[24-26]
	Amphibians	Frogs and newts	[27-30]
	Fishes	Spiny dogfish; mosquitofish and scaldfish	[31-33]
*Invertebrates*	Arthropods and annelids	Fruit flies, medflies, butterflies, mosquitoes, seed beetles, ants, bees, tarantulas, tea red spiders, copepods and the giant kidney-worms in the maned wolf	[34-44]

Footnote: An alternative form of longevity gender gap, i.e. males living longer than females, is also exhibited in a few animal taxa, especially the Aves [45].

Despite estrogen being described as a key factor contributing to the observed sex differences in telomere length (female > male) and longevity in animals, this hypothesis has never been validated using a suitable model system. The common animal models employed for aging studies, *Caenorhabditis elegans* and *Drosophila melanogaster*, have postmitotic cells predominantly in the somatic tissues of adult, making it unfeasible to investigate telomere-associated replicative senescence [46]. The conventional rodent models are not desirable for telomere- and estrogen- related aging studies. This is because telomeres of inbred rodents are extraordinarily long [47], making it difficult to study the effects of telomere erosion on aging and LGG in either short-term experiments or within a single generation. Moreover, the increased risk of breast and ovarian cancer development upon estrogen administration in rodents [48,49] complicates the study of the direct effects of estrogen on telomere length and longevity *in vivo*.

The small sized fish, Japanese medaka (*Oryzias latipes*), shares similar estrogen biology with mammals [50]. They undergo gradual senescence, increasing mortality rate with age [51-53] and progressive telomere shortening in most organs as it ages [54]. Our preliminary study further reveals female medaka live longer than the males. However, sex difference in telomere has never been reported in *O. latipes* or any teleost [45]. Using the medaka *O. latipes* as a model, the present study was designed to answer the fundamental question “Do sex differences (female > male) in both telomere length and longevity exist in fish?” Supported by the positive outcomes, we further examined age-associated changes in sex hormones, telomerase activity and telomere (length and attrition rate) in the liver (organ common in vertebrates) and gill (organ unique in fish), with an attempt to infer the mechanistic relationship among estrogen (sex), telomere and longevity in *O. latipes*. A theoretical ‘critical’ telomere length was derived in medaka liver for prediction of organismal senescence and mortality. Our findings also highlight a striking similarity between female *O. latipes* and women regarding their age-associated change in estrogen profile and telomere dynamic. The medaka *O. latipes* is therefore unique for studying the mechanisms of estrogenic (sex) influence on longevity in vertebrates, particularly human. To facilitate future research in this direction, we put forward a mathematical model that permits age conversion between medaka and human. Evidences provided herein strongly support that medaka fish is desirable for research in comparative biology of aging, unraveling the evidently conserved mechanism(s) of sex-dependent longevity in vertebrates.

## Results

### Sex-dependent lifespan of medaka

**Figure 1:**
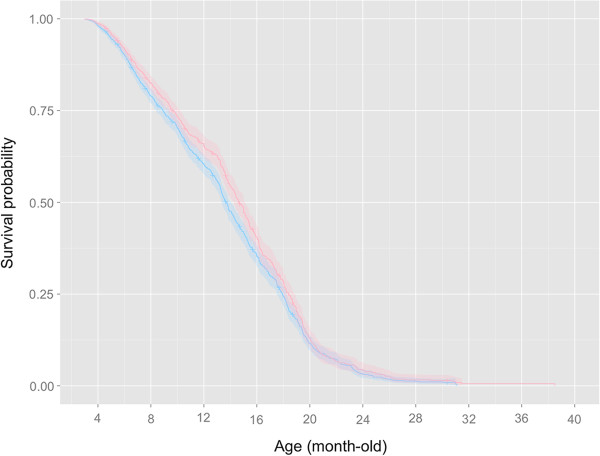
**Kaplan–Meier survival curves of the male (blue) and female (pink) *O. latipes *reared under optimal laboratory conditions (see Material and methods; N = 3117).** Confidence intervals (95%) of the survival rate are shown as semi-transparent colored bands. The survival probability of females is generally higher than that of males until 16 month-old, note the minor- to non-overlapping confidence intervals.

Male and female medaka reared under stable laboratory condition displayed significantly different survival profiles (Figure 1; logrank test: *χ*^2^ = 6.51, *df* =1, N = 3117, *p* = 0.011). Major discrepancy in survival probability between the two sexes was observed at 4 – 15 months old (Figure 1). The median life span of the males and females was 13.7 (95% CI: 13.3 – 14.1) months and 14.6 (95% CI: 14.3 – 15.2) months, respectively.

### Sex-dependent telomere length in the liver and gill of medaka during aging

**Figure 2:**
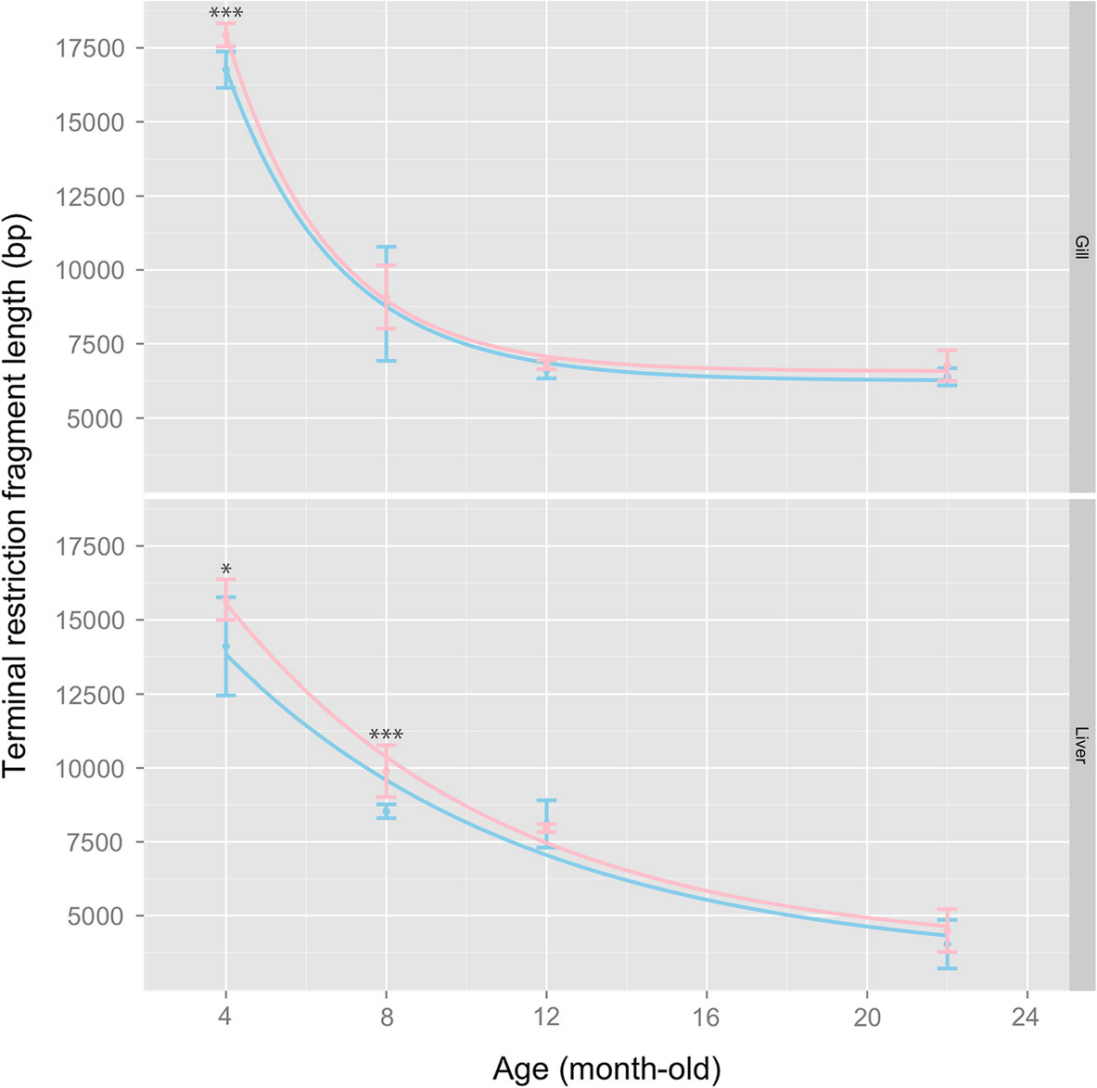
**Change of telomere length, as represented by the size of terminal restriction fragment (TRF; in bp), in male (blue) and female (pink) *O. latipes *during aging (upper: gill; lower: liver).** Shortening of organ TRF length across age in each sex was modeled by an exponential decay curve (see Materials and Methods). Error bars are standard error of mean (SEM). Asterisk indicates significant difference in TRF length between sexes at specific age: * = *p* < 0.05, *** = *p* < 0.001 after FDR-adjustment.

Telomere length (TL) represented by the mean terminal restriction fragment size (dubbed ‘TRF’ hereafter) in both gill and liver was not only age-dependent, but also sex-specific (Figure 2; two-way ANOVA interaction effect; Gill: *F*_3,70_ = 8.43, *p* < 0.001; Liver: *F*_3,72_ = 30.4, *p* = 0.034). Significant difference in TRF between sexes was observed in ‘younger’ individuals (i.e. gill: 4 months old; liver: 4–8 months old). Whenever significant difference in TRF was detected between two sexes, it was always longer in the females than in males (Figure 2).

### Organ-dependent telomere attrition rate in male and female medaka during aging

The age-associated decline of TRF, as observed in the gill and liver, grossly followed an exponential decay model (R^2^ ≥ 0.89). The rate of telomere attrition in the liver was significantly faster than that in the gill, and the telomere attrition rates were not sex-dependent (95% CI of λ: Liver ♂ = 1.67 – 2.55, ♀ = 1.75 – 2.13; Gill ♂ = 0.77 – 1.25, ♀ = 0.80 – 1.08). Besides, TRF in the gill was maintained in medaka from 12 months onwards; whereas in the liver, a continue reduction in TRF was observed across age for both sexes (Figure 2: top vs bottom panel).

### Sex-dependent telomerase activity in the liver and gill of medaka during aging

**Figure 3:**
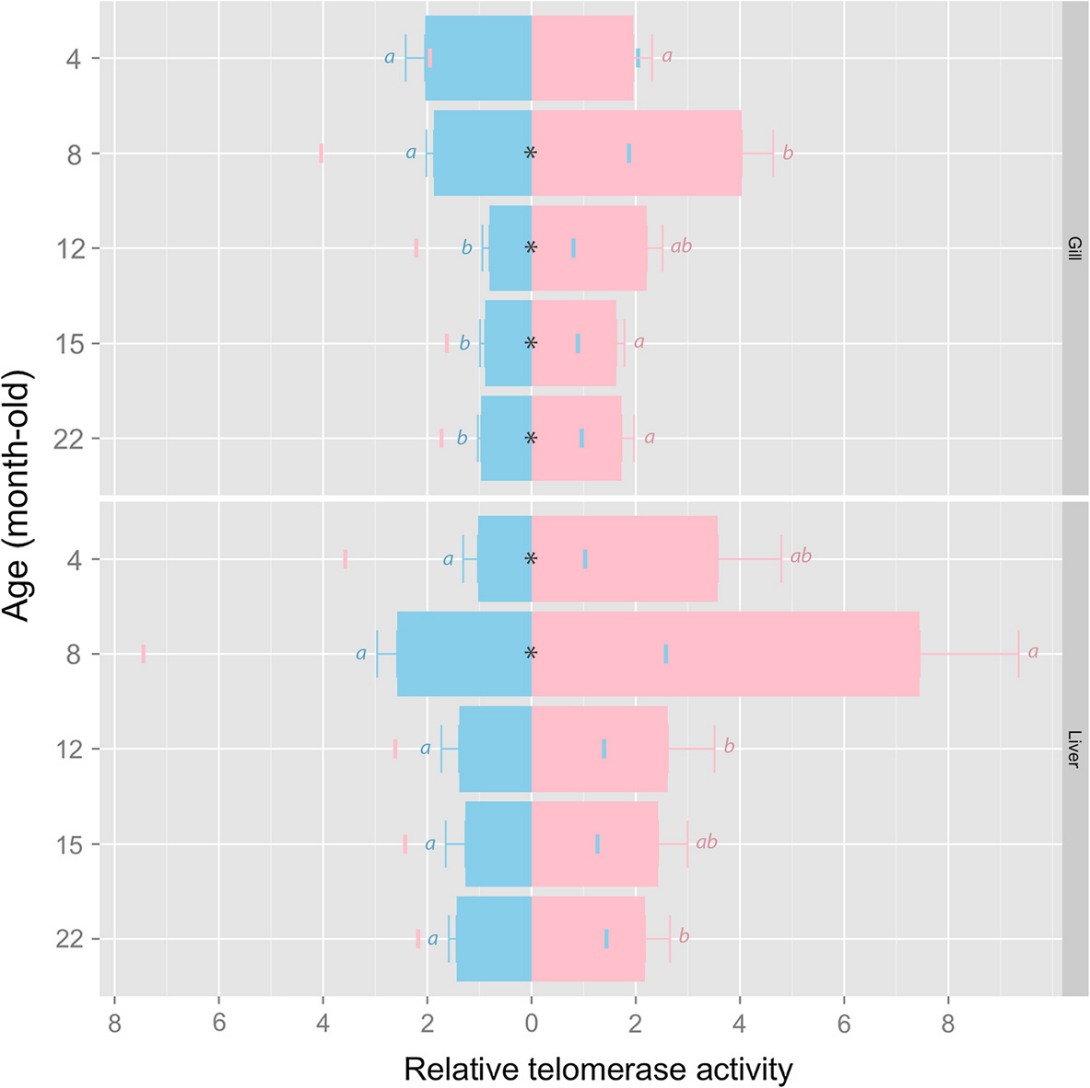
**Change of telomerase activity in male (blue) and female (pink) *O. latipes *during aging (upper: gill; lower: liver).** Relative telomerase activity is denoted by the bar length and the colored mark on the opposite side of the origin. Error bars are standard error of mean (SEM). Bars annotated with different alphabets are significantly different. Asterisk (*) indicates significant sex difference at that particular age (FDR-adjusted *p* < 0.05).

Telomerase activity was sex-specific and age-dependent in both gill (interaction effect: *F*_4,90_ = 4.15, *p* = 0.004) and liver (*F*_4,90_ = 7.75, *p* < 0.001). In gill, telomerase activity was significantly higher in females at 8–22 months old than in males of the same ages. By contrast, in the liver, 4–8 months old females showed higher telomerase activity than the male counterparts (Figure 3). Age dependent change in telomerase activity was much more prominent in females than in males (Figure 3: left vs right). In females, telomerase activity peaked in both gill and liver at 8 months old.

### Plasma sex hormone levels in male and female medaka during aging

**Figure 4:**
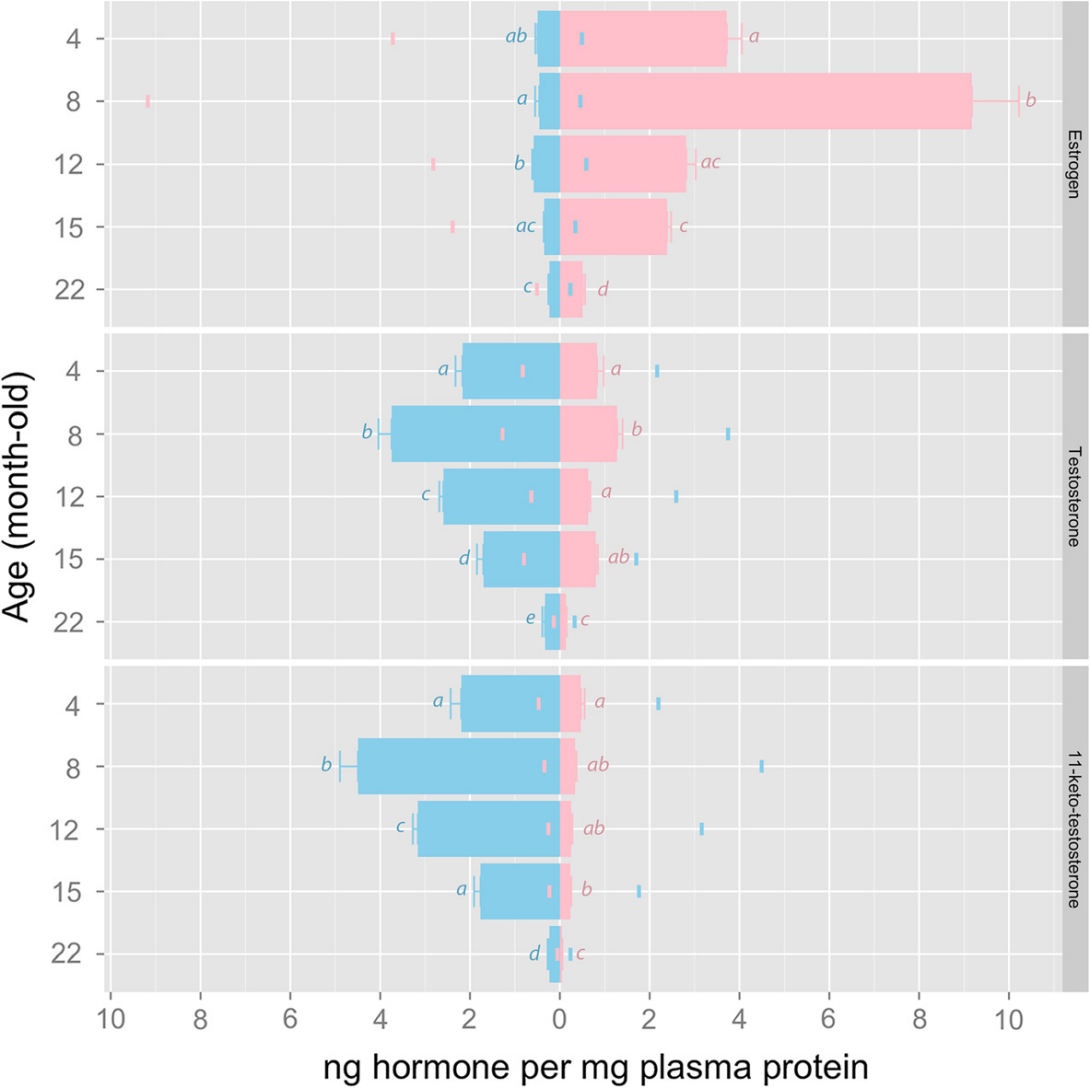
**Change of plasma sex hormones level in male (blue) and female (pink) *O. latipes *during aging (top: estrogen; middle: testosterone; bottom: 11-keto-testosterone).** Normalized hormone levels are denoted by the bar length and the colored mark on the opposite side of the origin. Error bars are standard error of mean (SEM). Bars annotated with different letters are significantly different. Statistical significant difference between the two sexes was detected for all three sex hormones at all age groups (all FDR-adjusted *p* < 0.05).

Statistical significant difference between males and females of the same age was detected in estradiol (E2), testosterone (T) and 11-keto-testosterone (11-KT) (all FDR-adjusted *p* < 0.05). Plasma E2 level in the females peaked at 8 months old and sharply declined thereafter, while that in the males was fairly constant. Similarly, plasma T and 11-KT levels peaked in males at 8 months old and gradually decreased with age, whereas their levels remained fairly stable in females across age. In both sexes, the levels of all three sex hormones in 22 months old *O. latipes* were significantly lower than fish of younger ages (4–15 months old) (*p* < 0.05) (Figure 4).

### Correlation between sex hormone levels, telomerase activity and telomere length

The relationships among levels of plasma sex hormones, telomerase activity and TRF were sex-specific and were much more evident in females (Table 2). In both gill and liver of females: (1) plasma E2 level was positively correlated with telomerase activity as well as TRF, and (2) T/11-KT also significantly correlated with TRF. Conversely, in males, (1) neither telomerase activity nor TRF correlated with E2 level, and (2) T/11-KT correlated with TRF in the liver only (not in gill) and at a lower correlation coefficients than that of the females. Spearman’s correlation analysis revealed no significant relationship between telomerase activity and TRF organ-wise and sex-wise in *O. latipes* (FDR-adjusted *p* > 0.05), except in the gill of males (ρ = 0.62, FDR-adjusted *p* < 0.001).

**Table 2:** **Spearman correlation between sex hormones and relative telomerase activity (TA) as well as telomere length (TL) in Japanese medaka *O. latipes***

		**Female**		**Male**	
		**TA**	**TL (TRF)**	**TA**	**TL (TRF)**
**Estrogen**	*Gill*	0.31*	0.51**	-	-
**(E2)**	*Liver*	0.34*	0.68**	-	-
**Testosterone**	*Gill*	-	0.47**	0.41*	-
**(TT)**	*Liver*	-	0.60***	-	0.41*
**11-keto testosterone**	*Gill*	-	0.54***	-	-
**(11-KT)**	*Liver*	-	0.71***	-	0.39*

Numerical figures are Spearman’s rank-order correlation coefficients (ρ) that are statistical significant. Asterisks represent FDR-adjusted *p*-values. *p < 0.05, **p < 0.01, ***p < 0.001, -: not significant.

### ‘Critical’ telomere length for medaka liver

**Figure 5:**
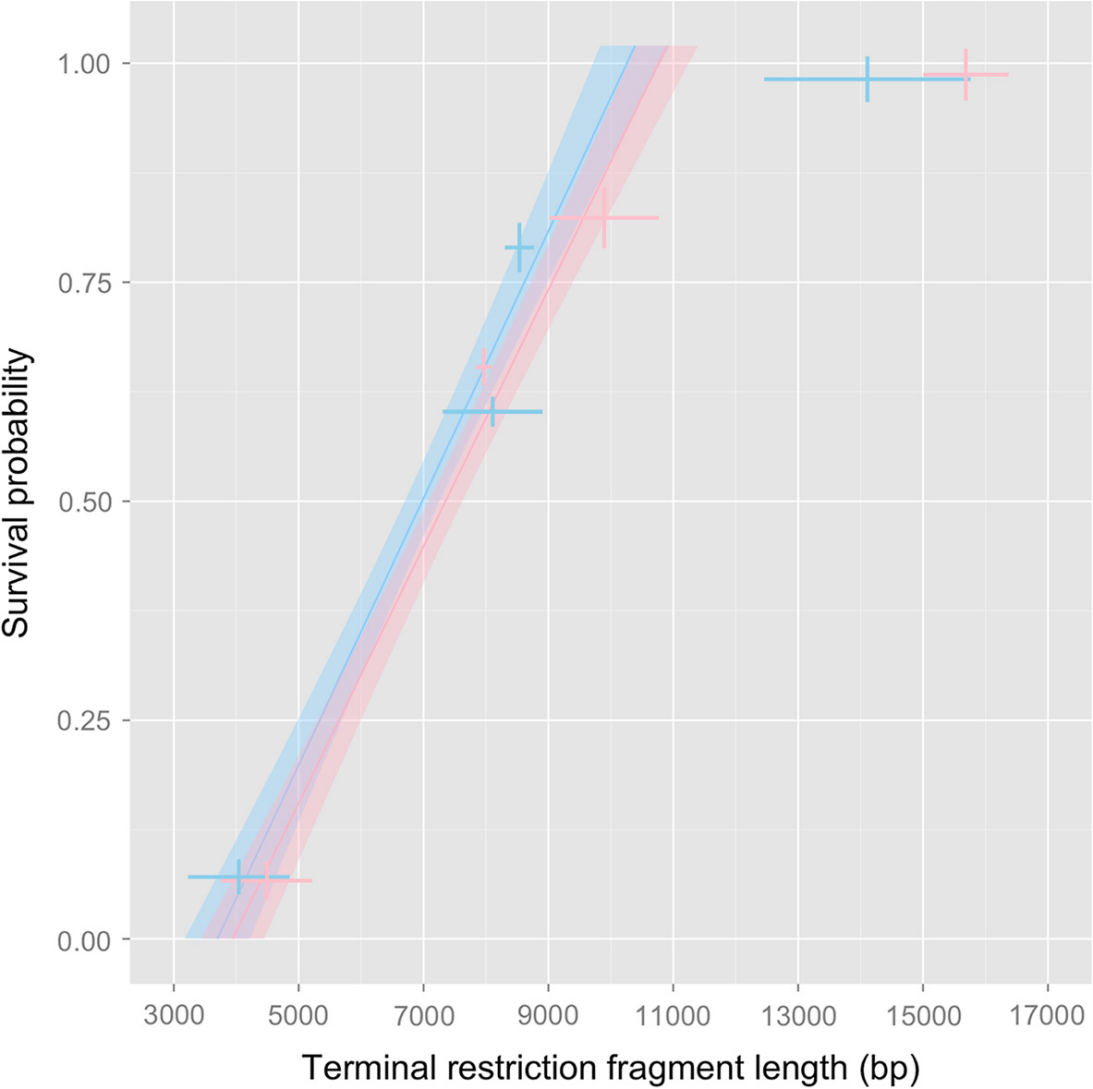
**A prediction model established to forecast survival probability from hepatic telomere length in *O. latipes*.** The survival probability is grossly proportional to the hepatic TRF length (R^2^ = 0.893). ‘Zero’ survival probability of male and female *O. latipes* is equivalent to a ‘critical’ length of ca. 3.8 kbp. Crosses are overlaid error bars (95% CI) for survival probability (vertical) and hepatic telomere length (horizontal).

Survival probability of individual fish was linearly related to the hepatic telomere length and reached almost 100% survival when TRF exceeded ~11 kb (Figure 5). Extrapolation of the TRF-survival relationship marks 0% survival probability (i.e. 100% mortality) when hepatic TRF decline to a mean ‘critical’ length of 3.8 kb and this was not sex-dependent (♂: 3.2 – 4.2 kb; ♀: 3.4 – 4.4 kb).

## Discussion

### Sex differences in telomere length and longevity exist in fish

This is the first report in teleost that females have a longer telomere length and lifespan than males. Our continued monitoring of over 3000 male and female Japanese medaka reared under optimal laboratory conditions for over three years produced sufficient and reliable data, supporting that a longer life expectancy in female *O. latipes* was concurred with a longer telomere in both the liver and gill. Further examination over a wide spectrum of fish species would be useful to verify the prevalence of these biological phenomena in primitive vertebrates.

### ‘Menopause’-like estrogen profile in female Japanese medaka during aging

Another novel finding arising from this study is the discovery of a natural drop of plasma E2 level in aging female *O. latipes*: E2 level was high in young females (4 months), peaked at mature females (8 months), followed by a significant decline in senior females (after 12 months). This is similar to the E2 profile from puberty to menopause in women [55]. In rodents, age-related decline of E2 level is not distinct, therefore ovariectomy is needed to mimic postmenopausal decline of E2 *in vivo*[56]. This invasive, artificial procedure could disturb body homeostasis, independent of the loss of estrogen [57]. In this regard, the *O. latipes* is unique over the rodent models for studying the estrogenic influence on telomere in ‘postmenopausal’ females without the necessity of ovariectomy.

### Potential mechanistic relationship between sex hormones, telomerase and telomere in medaka

Estrogen concentrations in plasma are useful to explain the sex-dependent telomere maintenance in *O. latipes*: when the females exhibited very high or peaked E2 levels at 4–8 months old, sex difference in telomere length (female > male) was the most distinct; whereas when E2 level declined in senior females, e.g. > 12 months old, such sex difference was correspondingly attenuated. It has been well proven using a variety of human cells [58] that estrogen enhances telomere maintenance by stimulating telomerase activity through ERE-dependent upregulation of TERT expression [11,12]. Ongoing study in our lab indicates the presence of functional EREs in *O. latipes* TERT promoter, suggesting this estrogen- and telomerase-dependent pathway also exists in *O. latipes*.

On the other hand, given a very weak link between testosterone/11-keto-testosterone and telomerase activity in both male and female *O. latipes* (Table 2), the regulatory effect of the two masculine hormones on telomere maintenance is likely independent of telomerase. This seems contradictory to previous *in vitro* studies showing that androgen could modulate hTERT expression and telomerase activity in prostate and hematopoietic cells [59-61]. The *in vivo* regulatory effect of androgen on telomerase and telomere maintenance in vertebrates warrants further investigation.

### Organ “critical” telomere length for prediction of survival probability

Earlier studies showed that human cells seldom possess mean TRF length shorter than 4 kb, and any further telomere attrition beyond this “critical” length was correlated with an induction of replicative senescence or cell death *in vitro*[61-63]. However, there was no report of similar “critical” telomere length in organs *in vivo* that could link to an induction of organismal senescence or mortality. Based on the temporal profile of telomere attrition in the liver of *O. latipes*, we have deduced a hypothetical *in vivo* ‘critical’ TRF length of approx. 4 kb, which is concurred with a zero survival probability of *O. latipes* (Figure 5). This theoretical *in vivo* ‘critical’ length in medaka liver is highly comparable to that for human cells, which may suggest senescence is triggered by a common telomere threshold. However, prior to making such a generalization, it is prudent to verify by using a variety of biological systems. Nevertheless, this ‘critical TRF’ in liver (not sex-dependent) marks a potential association between telomere attrition, organismal senescence and mortality of *O. latipes*. The potential of this ‘critical TRF’ as a novel molecular biomarker for predicting mortality in *O. latipes* warrants further testing and validation.

Furthermore, we recognize that ‘critical TRF’ could not be extrapolated for *O. latipes* gill which exhibited a level off of telomere length at old age. This also sheds light on the importance of multiple organ systems approach for a holistic understanding of telomere dynamics and aging progression in animals.

### Age conversion between medaka and human

**Figure 6:**
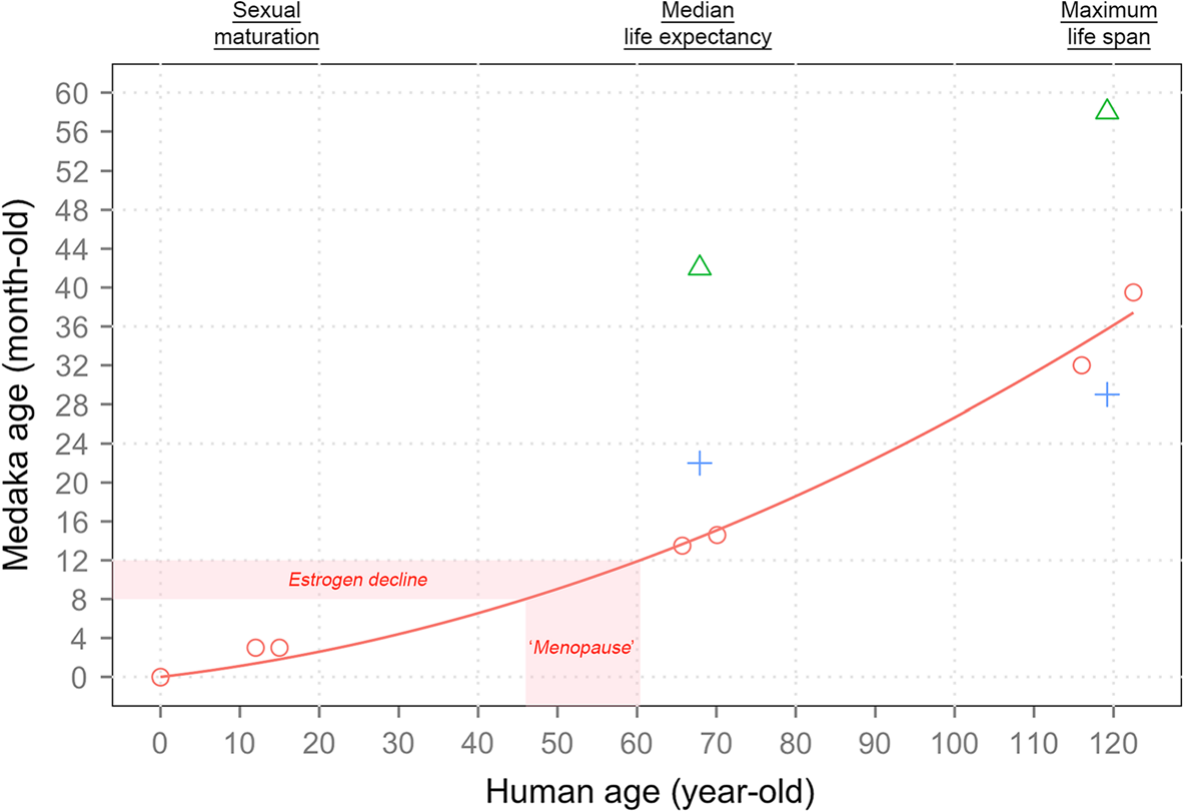
**An age conversion model for *O. latipes *(in months) and human (in years): *Age***_***medaka***_ **= 0.0954 ×** ***Age***_***human***_ **+ 0.0171 ×** ***Age***_***human***_^**2**^**.** Three major hallmarks: sexual maturation, median life expectancy and up-to-date record of maximum lifespan were used for regression. Red circles ○ represent data from the present study: sexual maturation (*O. latipes* : ca. 3 month-old; human: 12-15 year-old), median life expectancy (*O. latipes* : ♂ = 13.7 | ♀ = 14.6 months; human: world’s average ♂ = 65.7 | ♀ = 70.1 years) and up-to-date record of maximum lifespan (*O. latipes* : ♂ = 32.0 | ♀ = 39.5 months; human: ♂ Jiroemon Kimura = 116.1 | ♀ Jeanne Calment = 122.4 years). According to the regression curve (R^2^ = 0.996), the occurrence of estrogen decline in the female medaka (between 8 to 12 month-old) in our culture could be interpolated to human age of 46.0 to 60.4 year-old, which is akin to “menopause” in women. Previous aging data from [66] (blue cross +) and [54] (green triangle △) were overlaid for reference.

Our findings show that the initial TRF length (12 – 17 kb) of young Japanese medaka (4 months) was much shorter than mice (100 – 150 kb) [64] but similar to human (10 – 15 kb) [65]. Compare to rodent, the shorter telomere in medaka enables the study of the effects of telomere erosion on aging and LGG in a single generation. Interestingly, despite the similarity in initial and ‘critical’ TRF length between medaka and human, they exhibit distinct difference in lifespan (median in medaka: 14.3 months; in human, 65 years). The results imply one medaka-month could be equivalent to many human-years. To facilitate researchers to translate medaka age (in months) to human age (in years), we establish a mathematical model for age conversion (Figure 6) by using three key age hallmarks (sexual maturation age, the median lifespan and the maximum life span) reported for *O. latipes* to plot against the corresponding ages known for humans. Compare with other studies reported for *O. latipes* at these age hallmarks, our data were in line with [66], but different from [54]. This may be due to variation in husbandry conditions (e.g. indoor vs outdoor) in different laboratories. To validate and test the power of this conversion model, we interpolated 8- and 12- months old females, which encompassed a drastic decline in plasma E2 level after the peak at 8 months (Figure 6: highlighted band *above* the curve) to the corresponding ages in women (Figure 6: highlighted band *under* the curve). The results were found to be between 46 – 60 years old, which fully agree with the menopause age range in women [55]. This age conversion model is reliable and useful for future research on comparative biology of aging.

## Summary

The findings of this study strongly support the notion that *O. latipes* has advantages as a model for research on sex differences in telomere, aging and longevity because it exhibits: (i) longevity gender gap (female > male) and (ii) sex difference in telomere length (female > male). The natural, ‘menopause’-like drop of estrogen in females during aging renders medaka a unique model for studying the effect of ‘postmenopausal’ decline of estrogen on telomere and longevity without the need of invasive ovariectomy as required in rodents. Moreover, the direct effect of increased estrogen on telomere and longevity could be investigated in fish without the complication of breast cancer as reported in rodents. All these evidences highlight the strong potential of *O. latipes* as a unique non-mammalian model to advance research on estrogen- (sex-), telomere-associated aging and longevity in vertebrates. The *in vivo* critical length of medaka liver for prediction of organismal mortality and the age conversion model for medaka and human are useful tools for future research on comparative biology of aging using medaka. Overall, evidences provided herein highlight that the teleost *O. latipes* is significant for deciphering the ostensibly conserved mechanism(s) of sex-associated longevity in vertebrates.

## Material and methods

### Medaka culture

Orange-red, outbred line of Japanese medaka (*Oryzias latipes*) was originated from the Duke University, Molecular Aquatic Toxicology Laboratory. In 2008, fish were transferred and maintained as a colony in the City University of Hong Kong. Fertilized eggs were collected daily and reared until hatch as described in [67]. Fish that hatched in the same month were grouped as one cohort. Upon sexual maturation (approx. 3 month-old), males and females from the same cohort were randomly paired and transferred to aquarium tanks (39.5 L × 23.5 W × 27.5H cm) at a density of 15 pairs per tank. The fish were kept in the same set of tanks throughout their whole life.

All fish were kept under static condition of 26 ± 1°C, 7.2 ± 0.2 mg O_2_ L^-1^ and 14:10 hrs light-dark cycle. Half of the tank water is replaced with charcoal-filtered tap water every day. The fish were feed twice daily with Otohime β1 (Nisshin Co, Japan) and supplemented freshly hatched brine shrimp (*Artemia nauplia*) (Ocean Star International Lucky Brand, Utah, USA) 3 days per week. Fish health was closely monitored. Unhealthy fish, usually characterized by lack of appetite, inactivity, loss of golden-red color, hemorrhages, and/or external outgrowth, were promptly isolated into individual glass containers (diameter: 14.5 cm; height: 5 cm) containing 500 ml charcoal-filtered tap water. Every quarantined fish was reared under identical condition as described above and would not return to originated tank unless symptoms disappeared (‘recovered’) for at least 2 weeks. Records of stocking density and daily mortality of males and females in individual tank (hence individual cohort) were curated in SQLite 3 relational database management system (http://www.sqlite.org). These records were extracted for generating survival profiles and statistics.

### Fish sampling

Healthy fish at five different ages were sampled: 4- (‘young’), 8- (‘mature’), 12- (‘senior’), 15- (‘old’) and 22- (‘very old’) months. At each time point, 90 fishes (45 males and 45 females) were dissected. Fish were anesthetized in ice-cold aquarium water for 30 s, removed and measured for body length and weight. The fish was kept sedated by gently wrapping with paper towel fully soaked with ice-cold aquarium water. A cut was made to tail at 1–2 mm rostral to the caudal fin. Blood was drawn from the cut using P10 micropipette (Eppendorf, Hamburg, Germany) attached with heparinized pipette tips and instantly diluted with 8 μL double-distilled water to prevent clotting. The fish was then immediately decapitated. Gill and liver were isolated on ice bed and divided into two equal portions for parallel telomerase activity and telomeric length measurements. Blood and tissue samples from three fish of the same sex were pooled as one replicate (i.e. final sample number = 15 per sex per time-point). Pooled samples were snap-frozen in liquid nitrogen and stored at -80°C until further processing. Animal handling procedures as mentioned above were accepted by the Animal Ethics Committee, City University of Hong Kong.

### Telomere length measurement by Southern blot analysis

Genomic DNA was extracted from the livers and gills of adult male and female medaka using the DNeasy^®^ Blood and Tissue Kit (Qiagen, Hilden, Germany) according to the manufacturer’s instructions. For each assay, 3 μg genomic DNA was digested to completion with RsaI and HinfI (New England Biolabs, Massachusetts, USA) at 37°C overnight. The digested DNA was resolved by electrophoresis on a 1% agarose gel, run in parallel with a λ HindIII/EcoRI molecular marker (Fermentas, Burlington, Canada) and subsequently transferred to Hybond-XL membrane (GE Healthcare, Little Chalfont, United Kingdom) for overnight through capillary transfer. The membrane was saturated with ExpressHyb™ Hybridization Solution (Clontech, California, USA) at 42°C for 30 mins, and hybridized with 100 pmol of DIG-labeled oligonucleotide probes (TTAGGG)_5_ (Invitrogen, California, USA) in ExpressHyb™ Hybridization Solution (Clontech Laboratories, California, USA). The DIG-labeling was achieved by the use of DIG Oligonucleotide Tailing Kit, 2^nd^ generation (Roche Applied Science, Penzberg, Germany) according to the manufacturer’s instructions. After hybridization, the membrane was washed two times in 2X SSC buffer with 0.1% SDS at 25°C (5 mins each) and twice for 15 mins in 0.1X SSC with 0.1% SDS at 42°C. The washed membrane was blocked in 3% solution of non-fat milk powder (in phosphate buffered saline, PBS; pH 7.4) for 30 mins at room temperature, then incubated with 1:10000 anti-DIG, AP-conjugated antibodies (Roche). The membrane was then washed three times in PBS at RT and incubated with CDP-star (Roche) in darkness for 5 mins. Chemiluminescence was imaged by luminiscent image analyzer (Fujifilm LAS 4000, Tokyo, Japan) and saved lossless as TIFF. Telomere length was quantified in ImageJ as terminal restriction fragment (TRF) length following the procedure of [68].

### Telomerase activity assays by the real-time quantitative telomeric repeat amplification protocol (RTQ-TRAP)

Frozen tissues samples were thawed on ice bed and lysed in ice-cold CHAPS-containing lysis buffer (10 mM Tris–HCl, pH 7.5, 1 mM MgCl_2_, 1 mM EGTA pH 8.0, 0.5% CHAPS, 10% glycerol, 0.1 mM PMSF, 5 mM β-mercaptoethanol) (Sigma-Aldrich, Missouri, USA). The lysates were centrifuged at 16000 × g, 4°C for 30 mins. The supernatants were carefully transferred to new sterilized microfuge tubes. Protein concentration was determined using the Bradford protein assay (Bio-Rad, California, USA), with reference to standard curve constructed from serial dilutions of protein standard (bovine serum albumin, BSA; Sigma-Aldrich).

Telomerase activities in liver and gill samples of medaka were assessed by the RTQ-TRAP assay following the optimized protocol by [69]. Briefly, 40 ng of protein extract was added to a 25 μL reaction mixture of TRAP buffer [20 mM Tris-HCl (pH 8.3), 63 mM KCl, 3.5 mM MgCl_2_, 1 mM EGTA (pH 8.0), 0.1 mg/mL BSA, 0.005% Tween 20], 100 μM dNTPs, 1:25000 SYBR Green I dye, 10 nM ROX reference dye, 1.25 U HotStar Taq polymerase (Qiagen), 0.1 μg telomerase substrate primer (a.k.a. ‘TS’; 5′-AATCCGTCGAGCAGAGTT-3′; HPLC grade; Invitrogen), and 0.065 μg anchored return primer (a.k.a. ‘ACX’; 5′-GCGCGG(CTTACC)_3_CTAACC-3′; HPLC grade; Invitrogen). The reaction mixture was first incubated at 25°C for 30 mins to allow the telomerase in the protein extract to elongate the TS primer by adding TTAGGG-repeats. Next, the TRAP reaction was halted and the Taq polymerase was activated by heating at 95°C for 15 min. The activation was followed by 40 cycles of 95°C for 30 s, 60°C for 30 s, and 72°C for 60 s. Melting curve analysis was automatically carried out after completion of the 40^th^ cycle to verify the amplification specificity. Thermal cycling was conducted using the ABI 7500 Fast Real Time PCR System (Applied Biosystems, California, USA). Samples were run in triplicate. The C_q_ values were determined from semi-log amplification plots (log increase in fluorescence signal against cycle number). The relative telomerase activity was calculated by the 2^-ΔCq^ method [69].

### Sex hormone analysis

Blood samples were diluted with 300 μL double-distilled water and mixed with 2 mL of diethyl ether (Sigma-Aldrich). The mixture was vortexed for 10 seconds and centrifuged at 2000 × g for 10 mins. The upper, organic phase was transferred to glass centrifuge tube carefully. The extraction procedure was repeated 3 times to ensure complete recovery of the sex hormones. Diethyl ether was then evaporated under gentle stream of nitrogen gas (Hong Kong Oxygen Ltd, Hong Kong). Concentrations of estradiol, testosterones and 11-ketotestosterone were quantified using commercially available enzyme immunoassay kit (Cat.582251, Cat.582701, Cat.582751, respectively; Cayman Chemical, Michigan, USA) following the manufacturer’s recommended procedures. Plasma protein concentration of the whole blood was measured by the use of Bradford protein assay (Bio-Rad) and was used to normalize the concentration of sex hormones.

### Statistical analyses

All statistical analyses were performed in the R environment 2.15.1. Survival probability of male and female medaka across age was estimated using the Kaplan-Meier method adjusted for left-truncation (< 3 month-old) and right-censoring (up-to-date survivorship data at the time of this writing). Sex difference in longevity was indicated by contrasting the survival curves of the two sexes using logrank test. The age-dependent TRF length was modeled as exponential decay: *TRF*_*t*_ – *Plateau* = (*TRF*_0_ – *Plateau*) · *e*^–*λt*^ , where *TRF*_*t*_ is the modeled mean TRF length at age *t*, *TRF*_0_ is the extrapolated initial mean TRF length, *Plateau* is the horizontal asymptote, and *λ* is the rate constant. Model parameters were fitted using self-starting nonlinear least squares asymptotic regression model (R/nls in conjunction with SSasymp function).

Sex-dependency of the telomerase activity and telomeric length were tested using Student’s *t*-test. Age-dependent variation in plasma concentration of the three sex hormones’ level was illustrated by the use of one-way analysis of variance (ANOVA) followed by Tukey’s honestly significant difference (HSD) test. The relationship between telomere length and telomerase activity and the circulatory levels of sex hormones were illustrated by Spearman correlation analyses. Whenever applicable, the normality and homoscedascity assumptions for all parametric inferences were verified by the Shapiro-Wilk test and Levene’s test on median center, respectively. *p*-values from multiple comparisons were adjusted to control for false discovery rate (FDR) by use of the Benjamini and Hochberg procedure [70].

## Competing interests

The authors declare that they have no competing interests.

## Authors’ contributions

Experimental design: DWTA. Financial support: DWTA. Performed the experiments: GS. Data analysis and interpretation: NKMC, DWTA, GS, and BWPY. Manuscript preparation: GS, NKMC and DWTA. All authors read and approved the final manuscript.
